# Machine Learning Model Development and Validation for Predicting Outcome in Stage 4 Solid Cancer Patients with Septic Shock Visiting the Emergency Department: A Multi-Center, Prospective Cohort Study

**DOI:** 10.3390/jcm11237231

**Published:** 2022-12-05

**Authors:** Byuk Sung Ko, Sanghoon Jeon, Donghee Son, Sung-Hyuk Choi, Tae Gun Shin, You Hwan Jo, Seung Mok Ryoo, Youn-Jung Kim, Yoo Seok Park, Woon Yong Kwon, Gil Joon Suh, Tae Ho Lim, Won Young Kim

**Affiliations:** 1 Department of Emergency Medicine, College of Medicine, Hanyang University, Seoul 04763, Republic of Korea; 2 Research Coordinating Center, Konkok University Medical Center, Seoul 05030, Republic of Korea; 3 Department of Emergency Medicine, Korea University Guro Hospital, Seoul 08308, Republic of Korea; 4 Department of Emergency Medicine, Samsung Medical Center, Sungkyunkwan University School of Medicine, Seoul 06351, Republic of Korea; 5 Department of Emergency Medicine, Seoul National University Bundang Hospital, Seongnam 13620, Republic of Korea; 6 Department of Emergency Medicine, University of Ulsan College of Medicine, Asan Medical Center, Seoul 05505, Republic of Korea; 7 Department of Emergency Medicine, Yonsei University College of Medicine, Seoul 03722, Republic of Korea; 8 Department of Emergency Medicine, Seoul National University Hospital, Seoul 03080, Republic of Korea

**Keywords:** cancer patient, septic shock, machine learning, prognosis

## Abstract

A reliable prognostic score for minimizing futile treatments in advanced cancer patients with septic shock is rare. A machine learning (ML) model to classify the risk of advanced cancer patients with septic shock is proposed and compared with the existing scoring systems. A multi-center, retrospective, observational study of the septic shock registry in patients with stage 4 cancer was divided into a training set and a test set in a 7:3 ratio. The primary outcome was 28-day mortality. The best ML model was determined using a stratified 10-fold cross-validation in the training set. A total of 897 patients were included, and the 28-day mortality was 26.4%. The best ML model in the training set was balanced random forest (BRF), with an area under the curve (AUC) of 0.821 to predict 28-day mortality. The AUC of the BRF to predict the 28-day mortality in the test set was 0.859. The AUC of the BRF was significantly higher than those of the Sequential Organ Failure Assessment score and the Acute Physiology and Chronic Health Evaluation II score (both *p* < 0.001). The ML model outperformed the existing scores for predicting 28-day mortality in stage 4 cancer patients with septic shock. However, further studies are needed to improve the prediction algorithm and to validate it in various countries. This model might support clinicians in real-time to adopt appropriate levels of care.

## 1. Introduction

The incidence of all cancer types has increased worldwide and is a major public health burden [1]. Recent advances in cancer treatment have improved the overall survival. Nevertheless, there is an increased risk of critical illness requiring intensive care unit (ICU) management [2]. Reportedly, approximately 5.2% of patients develop a critical illness within 2 years after a cancer diagnosis and are admitted to the ICU [3]. The mortality rate in the ICU is reportedly 14.1%, and 24.6% of ICU patients die during their hospital stay [3]. Critical illness may make an important contribution to the overall cancer outcomes. In particular, the mortality rate is higher among patients admitted through the emergency department (ED) than elective admission.

Patients with cancer are at a more than 10-fold higher risk of sepsis than the general population. In addition, septic shock associated with cancer progression or chemoradiation therapy is a common life-threatening complication in stage 4 cancer patients [4]. The decision-making in invasive high-intensity care for stage 4 cancer patients with septic shock remains challenging, especially in EDs with a shortage of available ICU beds. The overutilization of an invasive ICU treatment in these circumstances often results in a more costly and invasive treatment without improving the outcomes [5,6]. Therefore, a reliable and clinically available prognostic score for advanced cancer patients with septic shock presenting at the ED is essential to improve the quality and efficiency of the ICUs care. However, traditional severity scoring systems such as the Sequential Organ Failure Assessment (SOFA) and Acute Physiology and Chronic Health Evaluation II (APACHE II) have not been validated in patients with advanced cancer and septic shock. Furthermore, the scores are not reliable at ED presentation because they are calculated on the worst parameters 24 h after admission [7,8]. To address this issue, a new predictive method is needed to improve the treatment of advanced cancer patients visiting the ED for sepsis and the use of the ICU. However, a prognosis prediction is difficult due to the heterogeneity and complexity of stage 4 cancer patients with septic shock. Machine learning (ML) algorithms have been published to improve the prognosis and occurrence prediction in other severe diseases such as sepsis, gastrointestinal bleeding, pneumonia, acute poisoning, and chronic obstructive pulmonary disease [9,10,11,12,13,14].

Therefore, the purpose of this study was to develop an ML model for stage 4 cancer patients with septic shock who visit the ED and to compare the diagnostic performance with those of the SOFA score, APACHE II score, and initial lactate level.

## 2. Materials and Methods

### 2.1. Study Population

This multi-center, retrospective, observational study used data from the Korean Shock Society (KoSS) septic shock registry between October 2016 and June 2019. The KoSS is a 11 university-affiliated hospital ED collaborative research network in South Korea, established in 2013, for improving the quality of the research, diagnosis, and management of sepsis [15]. The study included patients ≥ 19 years of age who met the inclusion criteria (evidence of refractory hypotension or hypoperfusion in patients with suspected or confirmed infection) [16,17]. Hypotension was defined as systolic blood pressure (SBP) < 90 mm Hg, mean arterial pressure < 70 mm Hg, or SBP decrease > 40 mm Hg. Refractory hypotension was defined as persistent hypotension despite the administration of a fluid challenge or the requirement of vasopressors to maintain a BP ≥ 90 mm Hg or mean arterial pressure ≥ 70 mm Hg [16,17]. Hypoperfusion was defined as the serum lactate level ≥ 4 mmol/L [18].

The exclusion criteria were patients who refused ICU management, patients who signed a “do not resuscitation” order before arrival at the ED or at the time of diagnosis, patients who met the inclusion criteria at 6 h after their ED arrival, patients who were transferred from other hospitals after their stabilization, and patients who were transferred directly to the ED of other hospitals. This study included only patients with stage 4 cancer who were registered in the septic shock database of a participating hospital. In stage 4 cancer, the disease has spread to other organs or parts of the body.

### 2.2. Ethics

This study complied with the principles of the 1964 Declaration of Helsinki. The Institutional Review Boards of Asan Medical Center [2015–1253], Korea University Anam Hospital [HRPC2016-184], Samsung Medical Center [SMC2015-09-057], Yonsei University College of Medicine Severance Hospital [4-2015-0929], Gangnam Severance Hospital [3-2015-0227], Seoul National University Bundang Hospital [B-1409/266-401], Seoul National University College of Medicine [J-1408-003-599], Seoul National University Boramae Medical Center [16-2014-36], Hallym University College of Medicine Gangnam Secred Heart Hospital [2015-11-142], Korea University Guro Hospital [KUGH15358-001], and Hanyang University Hospital [HYUH2015-11-013-007]) approved the study protocol. Informed consent was obtained from all subjects before the data collection if the patient was conscious, and the consent of the guardian was obtained otherwise.

### 2.3. Outcome and Data Collection

The endpoint selected to develop ML models was 28-day mortality. Death during hospitalization was confirmed by a record review, and death after discharge or transfer was confirmed by telephone. The study coordinator at each hospital confirmed 28-day mortality by a telephone interview. If the first call did not connect, an additional call was attempted, and there were no cases where 28-day mortality was not confirmed. The principal investigator of every site worked with a designated local research coordinator who was responsible for ensuring the accuracy of the data entry and verifying the records. A quality management committee of emergency physicians, local research coordinators, and investigators from every ED was established to monitor and review the data’s quality regularly. Members from the committee provided feedback on the results of the quality management process to the research coordinators and investigators, and doubts pertaining to the data were clarified either through the use of the system’s query function or directly via a telephone call. The data collection included the patient’s characteristics, clinical variables, and their laboratory results at presentation which were required to calculate the SOFA score, APACHE II score, and initial lactate level.

### 2.4. Machine Learning Model Development and Feature Analysis

During the pre-processing, any missing data were recorded as -1 because machine learning uses numeric data with no spaces as the input variables. Next, unnecessary data (attributes and feature selection methods) were excluded, leaving demographic characteristics, underlying diseases, blood test results, and other variables included in the 6 h bundle treatment after visiting the ED for analysis. However, variables that showed their worst value within 24 h of admission, such as the SOFA score and APACHEII score, were excluded from the machine learning model development. To develop an ML model for predicting the prognosis of advanced cancer patients, the dataset was randomly split in a 7:3 ratio (training: test) using stratified partitioning. Following initial resuscitation (6 h bundle therapy), all the identifiable variables (demographic, 6 h bundle therapy components, vital signs, laboratory variables, etc.) were used to develop the machine learning model. Under the stratified condition, the selected samples of each fold have proportions of class labels equal to those of the original dataset [19]. The best ML model and its optimal hyperparameters were determined in the training set using the stratified 10-fold cross-validation (in which the data set is divided into 10 folds, each of which is used for an internal validation, with the remaining 90% used for training to develop the model). The use of cross-validation and hyperparameter tuning for internal validation is considered a robust method for model evaluation before external validation on a separate data set and maximizes the potential performance of the ML model [20,21]. The hyperparameters of the model are optimized by a grid search that exhaustively considers all the parameter combinations. In the internal validation phase, the best hyperparameters are investigated to get the best performance. For example, the number of estimators from 100 to 1000 are investigated in balanced random forest (BRF). The detailed parameters are as follows: the parameters of LR-bw are C (0.001, 0.01, 0.1, 1, 10, and 100) and penalty (l1 and l2), the parameters of XGB-bw are the max_depth (3, 5, and 7) and subsample (0.6, 0,8, and 1.0), a parameter of RF-bw is the n_estimators (100, 200, 300, 400, 500, 600, 700, 800, 900, and 1000), a parameter of BBC is the n_estimators (100, 200, 300, 400, 500, 600, 700, 800, 900, and 1000), and a parameter of BRF is the n_estimators (100, 200, 300, 400, 500, 600, 700, 800, 900, and 1000).

To develop the ML model, a total of five classifiers were considered [10,22]. The dataset in this study showed the characteristics of an imbalanced dataset, because the number of data points were not balanced among the classes. Therefore, we used five weighted or balanced machine learning models in the machine learning model’s development. The three basic ML classifiers with balanced weights are considered (LR-bw, XGB-bw, and RF-bw). In addition, two ensemble classifiers that are designed for handling an imbalanced dataset are selected: the balanced bagging classifier and the balanced random forest classifier [23,24,25]. The best ML model was selected using the AUC and F1 score. The F1 score is a useful indicator for analyzing disproportionate data like ours (minor class prediction, 28-day mortality). After selecting the best ML model and its hyperparameters, the final ML was built using the training set. The performance of the ML was evaluated using the test dataset. The F1-score (F-measure) is a popular and suitable metric for an imbalanced classification [26,27]. It is widely used in many applications with an imbalanced dataset since it measures how well the classification model handled the minority class classification [28]. The F1 score is the harmonic mean of the precision and recall. Therefore, this metric balances a model in terms of the precision and recall.

For feature analysis, Shapley Additive exPlanations (SHAP) values were used. The SHAP values quantify the effects of the features on the outcome of the ML model [29]. In addition, the performance of the final ML model was compared with the existing severity scores of the SOFA, APACHE II, and initial lactate level. For the SOFA and APACHE II scores, the worst values obtained within 24 h of the ED visit were used. The calibration and discrimination of the final ML model and other scores were compared based on the calibration curve and AUC.

### 2.5. Statistical Analysis

The continuous variables were analyzed as the mean ± standard deviation or median with an interquartile range being appropriate, and the categorical variables were analyzed as the absolute or relative frequency. Student’s *t*-test and Mann–Whitney *U* test were used to compare the continuous variables, and the Chi-square test and Fisher’s exact test were used for the categorical variables. The discrimination and calibration of the final ML model and other scores were compared based on the AUC and calibration curve. The AUC was calculated and then compared using a 2-tailed nonparametric method [30].

A two-sided *p*-value < 0.05 was considered to be statistically significant. All the statistical analyses were performed using SPSS Statistics version 18 (SPSS Inc., Chicago, IL, USA) and R version 4.0.4 (R Foundation for Statistical Computing, Vienna, Austria). In addition, we used development software (Anaconda 3) for the ML on the Python platform. The Python version is 3.7 for windows (Python Software Foundation, Wilmington, DE, USA).

## 3. Results

### 3.1. Participant Characteristics

**Figure 1:**
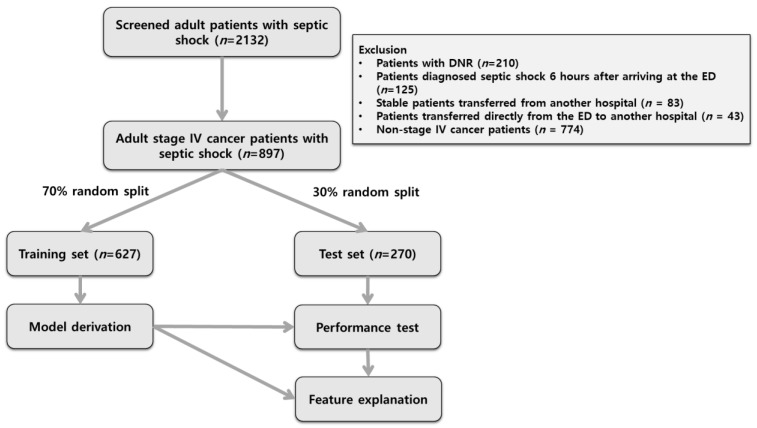
Patient selection flow diagram. DNR, do not resuscitate; ED, emergency department.

A total of 2132 patients were screened after excluding 210 patients with DNR orders (Figure 1). In addition, the cases diagnosed as septic shock 6 h or longer after arriving at the ED (*n* = 125) were excluded. Another 83 patients who were transferred from another hospital for septic shock but were stable were also excluded. In addition, 43 patients who were transferred directly from the ED to another hospital without admittance were excluded. After excluding 774 non-stage 4 cancer patients, a final 897 adult patients were included in the analysis. The dataset was split randomly in a 7:3 ratio (training: 627, test: 270). The baseline characteristics of 897 patients are shown in Table 1. The mean ages of the survivors and non-survivors were 65.6 and 66.9 years, respectively (*p* = 0.165). The proportion of males did not differ between survivors and non-survivors (61.8% vs. 64.6%, *p* = 0.482). The mean SBP of the non-survivors was significantly higher than that of survivors (99.5 vs. 94.9 years, *p* = 0.033). The median SOFA score for the non-survivors was significantly higher than for the survivors (10 vs. 7, *p* <0.001). The other characteristics between the survivors and non-survivors are presented in Table 1.

### 3.2. ML Model Development

Five weighted or balanced ML models were considered candidates for ML development because our data showed a disproportionate proportion of patients who died and survived. The best ML model in stratified 10-fold cross-validation was selected as the final ML model. The AUC of the BRF for 28-day mortality in the training set was 0.823 (95% confidence interval (CI): 0.782–0.864; Table S2), and the F1 score was 0.604 (95% CI: 0.548–0.66). The AUC of the BRF with a 10-fold validation in the training set is shown in Supplementary Figure S1. Based on this, the final chosen ML model was BRF. The AUC of the BRF for 28-day mortality in the test set was 0.826 (Table 2). The F1 score of the BRF was 0.64. The F1 score of the SOFA, APACHE II, and initial lactate was 0.321, 0.294, and 0.36, respectively.

### 3.3. Comparison of Diagnostic Performance of ML Model with Other Scores

**Figure 2:**
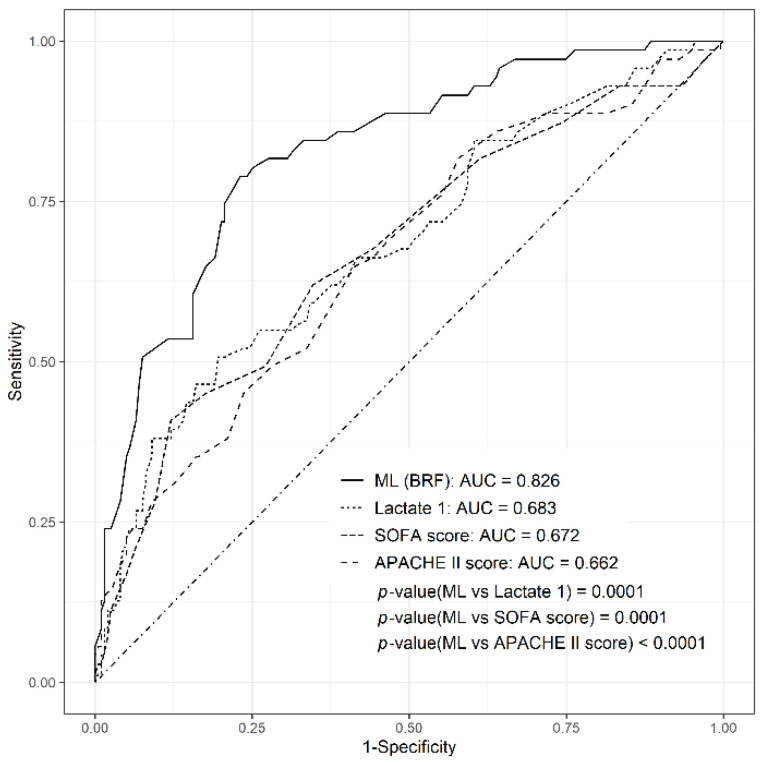
AUCs (areas under the curve) comparing the ML (machine learning) model with SOFA (Sequential Organ Failure Assessment), APACHE (Acute Physiology and Chronic Health Evaluation) II score, and initial lactate level for 28-day mortality in the test set.

The AUC of the BRF in the test set was 0.826. The AUCs of the SOFA, APACHE II, and initial lactate level in the test set were 0.672, 0.662, and 0.683, respectively (Figure 2). The AUC of the BRF was significantly higher than those of the SOFA, APACHE II, and initial lactate level (*p* = 0.0001, *p* < 0.0001, and *p* = 0.0001, respectively. After the Bonferroni correction on the test set, the AUC of the BRF model was significantly higher than the those of the SOFA and APACHE II score (Supplementary Table S3 and Figure S2).

**Figure 3:**
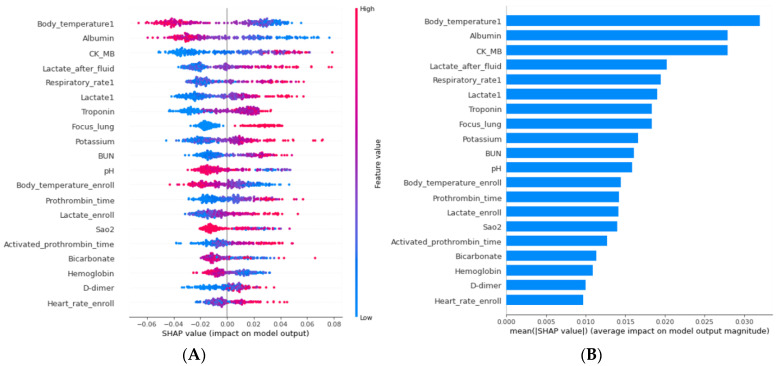
Summary of feature effects in the BRF model. (**A**) Overall effects of the top 20 variables. (**B**) Beeswarm plots showing the top 20 variables’ effects for all patients; Body_temperature1, initial body temperature; Lactate_after_fluid, lactate level following fluid administration; Respiratory_rate1, initial respiratory rate; Lactate1, initial lactate level; Focus_lung, lung as the site of infection; BUN, blood urea nitrogen level; pH, initial pH on arterial blood gas analysis; Body_temperature_enroll, body temperature when septic shock is recognized; Lactate_enroll, lactate level when septic shock is recognized; SaO2, initial arterial O2 saturation on arterial blood gas analysis; Heart_rate_enroll, heart rate when septic shock is recognized. All other variables are the first test values after the emergency department visit unless otherwise specified.

The top 20 most important variables for predicting 28-day mortality with BRF are summarized in Figure 3 with visible explanations across all the patients. The impacts of these features on the 28-day mortality were quantified by the Shapley values. Most of the variables were found to be related to the patient’s prognosis, similar to the results of previous studies, but the activated prothrombin time and potassium level were unexpected predictors. The initial body temperature had the highest overall (total 0.032) on the 28-day mortality, followed by the initial albumin level (0.028) and CK MB level (0.028). Calibration was evaluated with the plots of the predicted and observed probability. The ML model showed a better calibration compared with the SOFA score, APACHE II score, and initial lactate level in the test set (Figure S3). The ML model was closest to the ideal line and predicted the actual 28-day mortality rate well.

## 4. Discussion

In the present study, an ML (BRF) model to predict the prognosis of stage 4 cancer patients with septic shock was developed and validated. Several models showed a similar AUC to that of the BRF; however, BRF had the best F1 score. The AUC for the ML model to predict the 28-day mortality was superior to the traditional SOFA score, APACHE II score, and initial lactate level. The ML model showed a better calibration compared with the SOFA and APACHE II scores in both the training and test sets. The variables that had the greatest effect on the 28-day mortality were the initial body temperature, serum albumin level, and serum CK MB level. However, it is important to limit the application of the model because a score of F1 > 0.8 is necessary to consider a model as good. Moreover, the model is an insufficient basis for decisions on the orientation of the patients; it is important that a collegial discussion and exchanges with the families are also necessary to reach a shared decision.

To the best of our knowledge, this is the first study in which an ML model to predict the outcomes of stage 4 cancer patients with septic shock visiting the ED was developed and validated. Our study population was extracted from a multicenter study with a large sample size. The discrimination and calibration of ML were examined and compared with the pre-existing severity scores. We also used as many potential variables as possible in developing the ML model to influence the outcomes in patients with advanced cancer with septic shock.

Oncologic patients with sepsis and septic shock are admitted more frequently to the ICU than subjects from the general population. In addition, the 28-day mortality rate is higher in these patients [4,31]. However, the demand for the ICUs care usually exceeds the supply; therefore, the triage and allocation decisions for the ICUs care for critically ill patients are important [32]. Moreover, considering the high mortality rates associated with stage 4 solid cancer patients with septic shock visiting the ED, it is necessary to establish a risk score for providing professionals and families with more precise information for making the decision of whether to go ahead with an invasive procedure. It is also important to work on the early expression of patients’ wishes to be resuscitated or not. Scoring systems that predict the prognosis in cancer patients with septic shock have been investigated in several studies. The predictive values of the SOFA and APACHE II scores for the outcomes of patients with sepsis and septic shock were not satisfactory [33,34,35]. One ICU-based study reported that the AUC of the SOFA score for the in-hospital mortality was 0.69 in critically ill cancer patients with a suspected infection [33]. Another study reported that the AUC of the APACHE II score predicting in-hospital mortality in patients with sepsis or septic shock was 0.71 [36]. Compared to these studies using a similar study design, our ML model had an AUC greater than 0.8, although the primary outcome was different. In several studies, hypothermia was associated with increased mortality and organ failure in patients with severe sepsis [37,38]. Jonas et al. demonstrated that an elevated body temperature in patients admitted to the ED was associated with reduced mortality in patients with sepsis or septic shock admitted to the ICU [39]. However, a fever above 39.5 °C was associated with an increased mortality rate [40]. In several sepsis studies, the serum albumin level was associated with increased mortality [41,42,43]. An elevated troponin level was correlated with a greater degree of left ventricular dysfunction, illness severity, and mortality [44,45]. In a meta-analysis, 61% of subjects with elevated troponin had a twofold increased risk of death compared with patients with undetectable troponin [46]. In our machine learning model, troponin had a lower prognostic contribution than CK MB, but similarly to previous studies, elevated myocardial enzyme levels in sepsis and septic shock were highly associated with a poor prognosis. To recognize the occurrence of sepsis or predict the prognosis in the early stages while the patient is in the ward or ICU, research using ML is being actively conducted [11,47].

The present study had several limitations. First, it only included single-country data and a relatively small sample size, although multi-center prospectively collected registry data were used. Therefore, the results might not be representative of the general population. Unlike other studies using ML models, in the present study, the diagnostic performance in the test set is slightly higher than the diagnostic performance of the training set. Although we randomly divided the data into the training set and the test set, since we used patients with similar characteristics, we think that the similarity between the training set and the test set still exists and the diagnostic performance of the test set may have been higher as a result of chance. In future work, the performance of our ML model must be tested using independent cohort data for an accurate evaluation. The data used is from a registry, which inherently limits the applicability of such an algorithm with real-world data. Therefore, ML models should be prospectively validated using larger samples and a variety of real-world data from many countries and ethnicities. Second, cancer-related characteristics such as the treatment modality (e.g., surgery, radiotherapy, and chemotherapy), performance status, and response to therapy were not examined. These factors are known to be associated with the outcomes. However, in recent studies, cancer-related characteristics were not associated with mortality [48,49]. The accurate evaluation of the performance status is challenging in the ED due to subjectivity and irreproducibility [50,51]. Third, there may be unmeasured confounders that can affect the results. Our registry was for the general population with septic shock and was not cancer-specific. Therefore, the possibility of unmeasured variables, especially cancer-specific prognostic factors, cannot be excluded. Nevertheless, many variables associated with the outcome of patients with septic shock were included. Lastly, although 10 hospitals participated in the surviving sepsis campaign for the septic shock treatment, there might be differences in the treatment strategies between hospitals.

## 5. Conclusions

The predictive performance of the ML model was satisfactory to predict 28-day mortality in stage 4 cancer patients with septic shock. The ML model outperformed the pre-existing prediction scores, such as the SOFA, APACHE II, and initial lactate level. We revealed 20 important variables that significantly affected the prediction model. This model might support clinicians in real-time to adopt an appropriate level of care in terms of the chance of survival. However, the level of treatment should not be determined by the ML model alone without a co-operative discussion or exchange with the families. Additionally, further studies are needed to improve the prediction algorithm and to validate it in various countries.

## Figures and Tables

**Table 1:** Comparison of characteristics of survivors and non-survivors between October 2016 and June 2019 in 11 university-affiliated hospital emergency departments in South Korea.

	Total Patients	Survivors (*n* = 660)	Non-Survivors (*n* = 237)	*p*-Value
Age, mean ± standard deviation, y	66 ± 11.5	65.6 ± 11.2	66.9 ± 12.4	0.165
male, no. (%)	561 (62.5%)	408 (61.8%)	153 (64.6%)	0.482
Vital signs, mean ± standard deviation				
Initial systolic blood pressure, mmHg	96.1 ± 27.1	94.9 ± 26.2	99.5 ± 26.1	0.033
Initial diastolic blood pressure, mmHg	59.5 ± 18.5	58.82 ± 17.6	61.2 ± 20.7	0.11
Initial heart rate, beats per min	114 ± 25.1	113.9 ± 24.9	114.3 ± 25.6	0.818
Initial respiratory rate, breaths per min	21.4 ± 4.9	20.8 ± 4.1	23.1 ± 6.4	<0.001
Initial body temperature, °C	37.8 ± 1.3	38 ± 1.3	37.2 ± 1.2	<0.001
Laboratory findings, median (interquartile range)				
White blood cells, 10^3^/mm^3^	8.1 (3.0–15.4)	7.9 (3.1–15)	8.9 (2.5–18)	0.165
Hb, g/dL	10.2 (8.6–11.7)	10.2 (8.7–11.6)	9.9 (8.5–11.8)	0.604
Platelets, 10^3^/mm^3^	143 (71–232)	147 (78–238)	129 (61–226)	0.043
Albumin, g/dL	2.8 (2.3–3.2)	2.8 (2.5–3.3)	2.5 (2.1–3)	<0.001
Blood urea nitrogen, mg/dL	25 (17–38)	23 (16.4–34.5)	31 (20.3–47.2)	<0.001
Creatinine, mg/dL	1.3 (0.9–1.9)	1.2 (0.9–1.9)	1.4 (0.9–2.2)	0.023
C-reactive protein, mg/dL	12.8 (6.1–22.1)	12.5 (5.5–21.6)	13.6 (6.9–25.1)	0.137
Lactate, mmol/L	3.6 (2–5.5)	3.3 (1.9–5)	5 (3.1–7.7)	<0.001
Cancer type, no. (%)				
Stomach	55 (6.1%)	39 (5.9%)	16 (6.8%)	0.753
Colorectal	63 (7%)	53 (8%)	10 (4.2%)	0.054
Liver	113 (12.6%)	90 (13.6%)	23 (9.7%)	0.138
Biliary	85 (9.5%)	67 (10.2%)	18 (7.6%)	0.301
Pancreas	88 (9.8%)	70 (10.6%)	18 (7.6%)	0.204
Lung	151 (16.8%)	81 (12.3%)	70 (29.5%)	<0.001
Gynecologic	82 (9.1%)	67 (10.2%)	15 (6.3%)	0.088
Urologic	78 (8.7%)	65 (9.8%)	13 (5.5%)	0044
Other	182 (20.3%)	128 (19.4%)	54 (22.8%)	0.3
Infection focus, no. (%)				
Lung	256 (28.5%)	151 (22.9%)	105 (44.3%)	<0.001
Urinary tract	146 (16.3%)	113 (17.1%)	33 (13.9%)	0.262
Gastrointestinal	163 (18.2%)	115 (17.4%)	48 (20.3%)	0.377
Hepatobiliary	257 (28.7%)	210 (31.8%)	47 (19.8%)	0.001
Bone soft tissue	20 (2.2%)	16 (2.4%)	4 (1.7%)	0.616
Others	35 (3.9%)	32 (5.6%)	3 (1.6%)	0.026
Comorbidities, no. (%)				
Hypertension	281 (31.3%)	207 (31.4%)	74 (31.2%)	1.000
Diabetes mellitus	207 (23.1%)	152 (23%)	55 (23.2%)	1.000
Cardiac disease	77 (8.6%)	47 (7.1%)	30 (12.7%)	0.011
Cerebrovascular accident	39 (4.3%)	17 (3%)	14 (7.3%)	0.011
Chronic lung disease	60 (6.7%)	37 (6.5%)	17 (8.9%)	0.328
Chronic renal disease	33 (3.7%)	28 (4.2%)	5 (2.1%)	0.161
Chronic liver disease	78 (8.7%)	62 (9.4%)	16 (6.8%)	0.23
SOFA score, median (interquartile range)	8 (6–10)	7 (5–10)	10 (7–13)	<0.001
APACHE II score, median (interquartile range)	20 (15–26)	19 (14–24)	24 (19–32)	<0.001
Outcomes, no. (%)				
28-day mortality	237 (26.4%)			
Time to death, day	8 (2–7)			
Vasopressor	653 (72.8%)	493 (86%)	160 (83.8%)	0.477
Renal replacement treatment	80 (8.9%)	30 (4.5%)	50 (21.1%)	<0.001
Intensive care unit admission	412 (45.9%)	324 (49.1%)	88 (37.1%)	0.002
Mechanical ventilation	192 (21.4%)	88 (13.3%)	104 (43.9%)	<0.001
DNR in intensive care unit or general ward	246 (27.4%)	80 (12.1%)	166 (70%)	<0.001

These data were measured or confirmed immediately after the emergency department visit. APACHE II, Acute Physiology and Chronic Health Evaluation II; DNR, do not resuscitate; SOFA, Sequential Organ Failure Assessment.

**Table 2:** Comparison of machine learning models for predicting 28-day mortality in the test set between October 2016 and June 2019 in 11 university-affiliated hospital emergency departments in South Korea.

	AUC (95% CI)	F1 Score
LR-bw	0.763 (0.696–0.831)	0.596
XGB-bw	0.779 (0.717–0.841)	0.562
RF-bw	0.811 (0.755–0.868)	0.292
BBC	0.796 (0.738–0.855)	0.605
BRF	0.826 (0.77–0.881)	0.64
SOFA score	0.672 (0.596–0.748)	0.321
APACHE II score	0.662 (0.587–0.736)	0.294
Initial lactate	0.683 (0.609–0.757)	0.36

AUC, area under the curve; APACHE II, Acute Physiology and Chronic Health Evaluation; BBC, balanced bagging classifier; BRF, balanced random forest; CI, confidence interval; LR-bw, logistic regression with balanced weight; ML, machine learning; SOFA, Sequential Organ Failure Assessment; RF-bw, random forest with balanced weight; XGB-bw, XGB with balanced weight.

## Data Availability

The study data will be made available upon request to the corresponding author.

## Supplementary Materials

The following supporting information can be downloaded at: https://www.mdpi.com/article/10.3390/jcm11237231/s1, Table S1. Comparison of the training and test sets. Table S2. Comparison of machine learning models for predicting 28-day mortality in training sets. Table S3. Comparison of ML models for predicting 28-day mortality in test sets. Figure S1. The AUC of BRF with 10-fold validation in the training set. Abbreviations: AUC—area under the curve; BRF—balanced random forest. Figure S2. The AUC of ML models for predicting 28-day mortality in the test set. Abbreviations: AUC—area under the curve; BBC—balanced bagging classifier; BRF—balanced random forest classifier; CI—confidence interval; LR-bw—logistic regression with balanced weight; ML—machine learning; RF-bw—random forest classifier with balanced weight; XGB-bw—XGB classifier with balanced weight. Figure S3. Calibration curve of the ML model, SOFA, APACHE II and initial lactate level for 28-day mortality in the test set. Abbreviations: BRF—balanced random forest; ML—machine learning; SOFA—Sequential Organ Failure Assessment; APACHE—Acute Physiology and Chronic Health Evaluation.
