# Supplementary material for: Machine Learning Model Development and Validation for Predicting Outcome in Stage 4 Solid Cancer Patients with Septic Shock Visiting the Emergency Department: A Multi-Center, Prospective Cohort Study
Source: J Clin Med. 2022 Dec 5;11(23):7231. doi: 10.3390/jcm11237231 (PMC9737041; doi:10.3390/jcm11237231)
Supplement: Supplementary file 1 [file jcm-11-07231-s001.zip › jcm-2006623-supplementary.pdf]

Supplementary Table S1. Comparison of the training and test sets.

	Training set (n = 627)	Test set (n = 270)	*p*-Value
Age, mean ± standard deviation, y	66.3 ± 11.6	65.1 ± 11.4	0.152
Male, no. (%)	289 (63.5%)	119 (60.7%)	0.537
Vital signs, mean ± standard deviation			
Initial systolic blood pressure, mmHg	96 ± 27.5	96.5 ± 26.1	0.775
Initial diastolic blood pressure, mmHg	59.2 ± 18.1	60 ± 19.3	0.547
Initial heart rate, beats per min	114.2 ± 25.2	113.6 ± 24.7	0.727
Initial respiratory rate, breaths per Min	21.4 ± 5.1	21.4 ± 4.5	0.926
Initial body temperature, °C	37.7 ± 1.3	37.8 ± 1.2	0.717
Laboratory findings, median (interquartile range)			
White blood cell, $10^3/mm^3$	8.1 (3.1–15)	8.2 (2.8–16)	0.743
Hemoglobin, g/dL	10.3 (8.6–11.7)	9.9 (8.4–11.6)	0.077
Platelets, $10^3/mm^3$	147 (73–238)	135 (68–221)	0.486
Albumin, g/dL	2.8 (2.3–3.2)	2.8 (2.4–3.2)	0.57
Blood urea nitrogen, mg/dL	25 (17.4–39)	23.3 (17.2–35.3)	0.304
Creatinine, mg/dL	1.3 (0.9–2)	1.2 (0.9–1.8)	0.45
C-reactive protein, mg/dL	12.8 (5.9–22.4)	12.7 (6.1–21.7)	0.562
Lactate, mmol/L	3.6 (2–5.5)	3.7 (2–5.6)	0.848
Cancer type, no. (%)			
Stomach	42 (6.7%)	13 (4.8%)	0.294
Colorectal	41 (6.5%)	22 (8.1%)	0.395
Liver	84 (13.4%)	29 (10.7%)	0.276
Biliary	57 (9.1%)	28 (10.4%)	0.619
Pancreas	63 (10%)	25 (9.3%)	0.807
Lung	106 (16.9%)	45 (16.7%)	1.000
Gynecologic	55 (8.8%)	27 (10%)	0.614
Urologic	57 (9.1%)	21 (7.8%)	0.606
Other	122 (19.5%)	60 (22.2%)	0.366
Infection focus, no. (%)			
Lung	174 (27.8%)	82 (30.4%)	0.468
Urinary tract infection	108 (17.2%)	38 (14.1%)	0.278
Gastrointestinal	110 (17.5%)	53 (19.6%)	0.509
Hepatobiliary	187 (29.8%)	70 (25.9%)	0.26
Bone soft tissue	17 (2.7%)	3 (1.1%)	0.149
Others	27 (4.3%)	8 (3%)	0.359
Comorbidities, no. (%)			
Hypertension	208 (33.2%)	73 (27%)	0.072
Diabetes mellitus	140 (22.3%)	67 (24.8%)	0.437
Cardiac disease	52 (8.3%)	25 (9.3%)	0.697
Cerebrovascular accident	25 (4%)	14 (5.2%)	0.475
Chronic lung disease	43 (6.9%)	17 (6.3%)	0.774
Chronic renal disease	24 (3.8%)	9 (3.3%)	0.848
Chronic liver disease	56 (8.9%)	22 (8.1%)	0.796
SOFA score, no. (%)	8 (6-10)	8 (6-11)	0.897
APACHE II score, no. (%)	20 (15-26)	19 (14-26)	0.719

Outcomes, no. (%)			
Vasopressor	449 (84.2%)	204 (88.3%)	0.148
28-day mortality, No. (%)	166 (26.5%)	71 (26.3%)	1.000
Intensive care unit admission	292 (46.6%)	120 (44.4%)	0.56
Mechanical ventilation	127 (20.3%)	65 (24.1%)	0.214

APACHE II—Acute Physiology and Chronic Health Evaluation II; SOFA—Sequential Organ Failure Assessment.

Supplementary Table S2. Comparison of machine learning models for predicting 28-day mortality in training sets.

	**LR-bw**	**XGB-bw**	**RF-bw**	**BBC**	**BRF**
AUC (95% CI)	0.784 (0.743–0.826)	0.789 (0.744–0.835)	0.826 (0.788–0.863)	0.815 (0.782–0.849)	0.823 (0.782–0.864)
F1 score	0.552 (0.5–0.604)	0.583 (0.518–0.648)	0.392 (0.299–0.485)	0.572 (0.513–0.631)	0.604 (0.548–0.66)

AUC—area under the curve; BBC—balanced bagging classifier; BRF—balanced random forest classifier; CI—confidence interval; LR-bw—logistic regression with balanced weight; ML—machine learning; RF-bw—random forest classifier with balanced weight; XGB-bw—XGB classifier with balanced weight.

Supplementary Table S3. Comparison of ML models for predicting 28-day mortality in test sets.

Variable 1	Variable 2	Comparison AUC p-value	Bonferroni corrected p-value*
LR_bw	SOFA score	0.0389	0.5836
LR_bw	APACHE II score	0.0231	0.3472
LR_bw	Lactate1	0.0769	>.9999
XGB_bw	SOFA score	0.0104	0.1561
XGB_bw	APACHE II score	0.0088	0.1317
XGB_bw	Lactate1	0.0267	0.4007
RF_bw	SOFA score	0.0002	0.0032
RF_bw	APACHE II score	0.0002	0.0030
RF_bw	Lactate1	0.0005	0.0070
Balanced Bagging Classifier	SOFA score	0.0014	0.0217
Balanced Bagging Classifier	APACHE II score	0.0013	0.0195
Balanced Bagging Classifier	Lactate1	0.0026	0.0396
Balanced Random Forest	SOFA score	0.0001	0.0011
Balanced Random Forest	APACHE II score	<.0001	0.0006
Balanced Random Forest	Lactate1	0.0001	0.0022

* Adjustment for 15 pairs of tests

AUC—area under the curve; BBC—balanced bagging classifier; BRF—balanced random forest classifier; CI—confidence interval; LR-bw—logistic regression with balanced weight; ML—machine learning; RF-bw—random forest classifier with balanced weight; XGB-bw—XGB classifier with balanced weight.

Figure S1. The AUC of BRF with 10-fold validation in the training set. Abbreviations: AUC—area under the curve; BRF—balanced random forest.

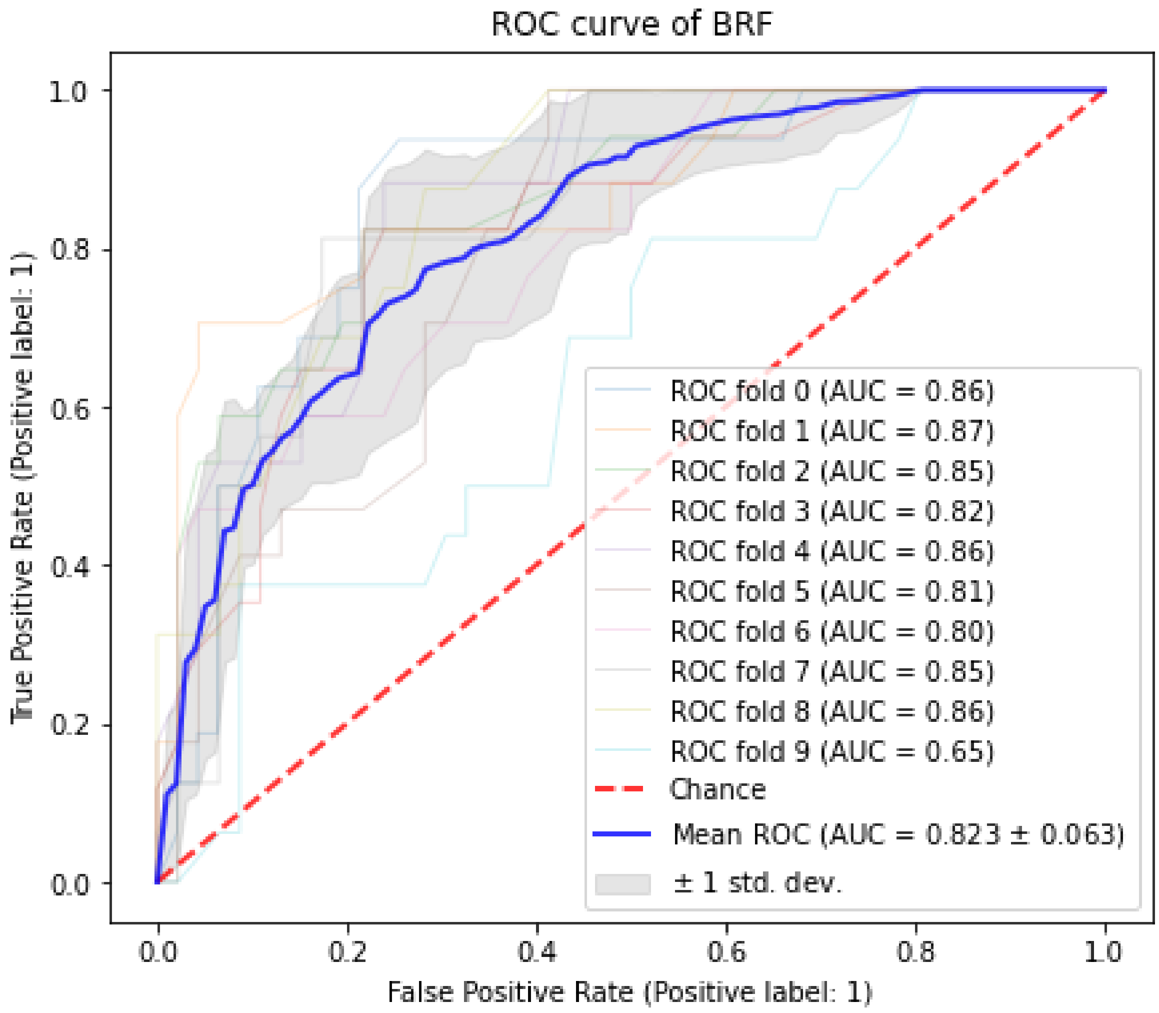

Figure S2. The AUC of ML models for predicting 28-day mortality in the test set. Abbreviations: AUC—area under the curve; BBC—balanced bagging classifier; BRF—balanced random forest classifier; CI—confidence interval; LR-bw—logistic regression with balanced weight; ML—machine learning; RF-bw—random forest classifier with balanced weight; XGB-bw—XGB classifier with balanced weight.

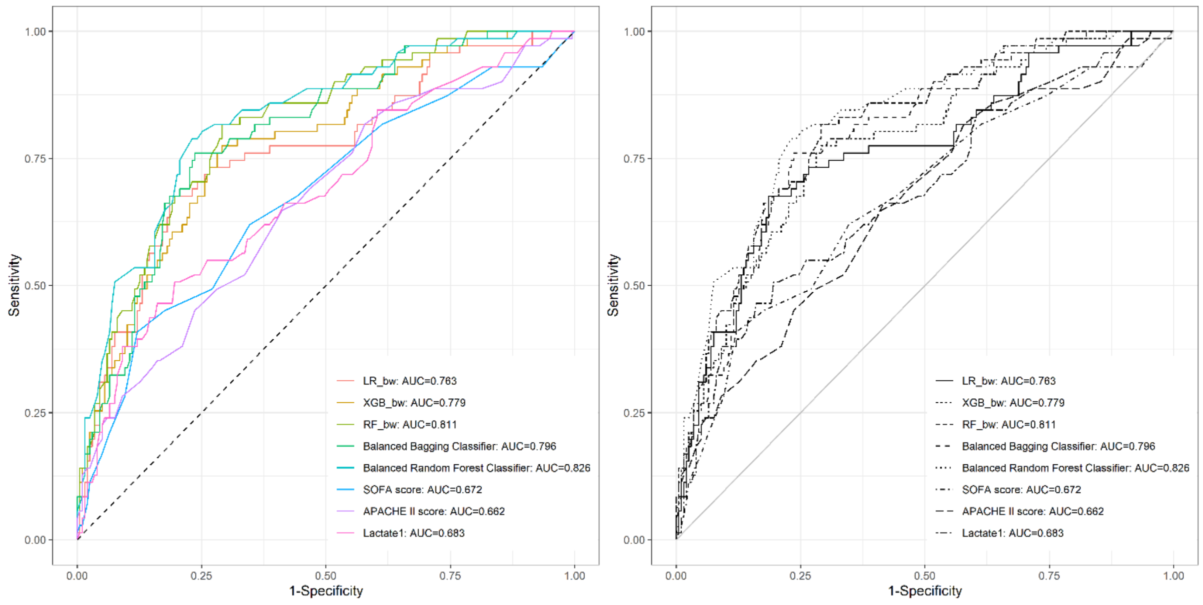

Figure S3. Calibration curve of the ML model, SOFA, APACHE II and initial lactate level for 28-day mortality in the test set. Abbreviations: BRF—balanced random forest; ML—machine learning; SOFA—Sequential Organ Failure Assessment; APACHE—Acute Physiology and Chronic Health Evaluation.

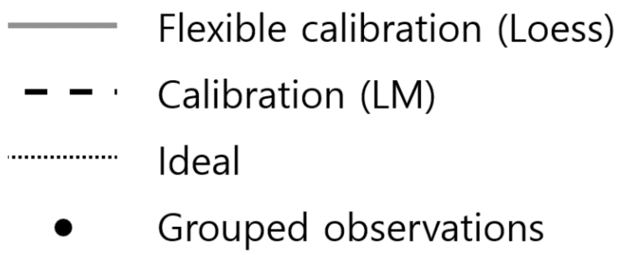

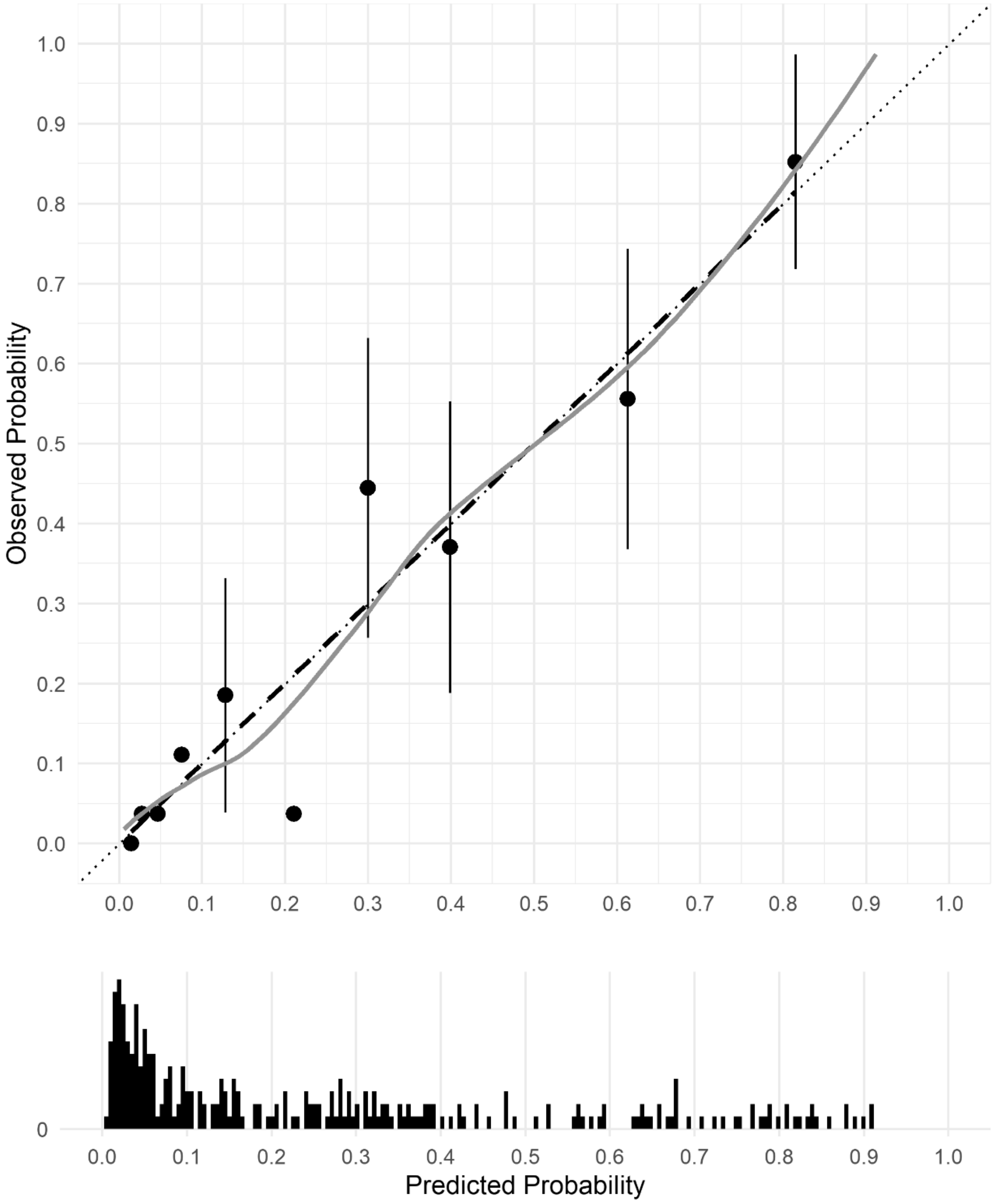

Observed Probability
Predicted Probability
1.0
0.9
0.8
0.7
0.6
0.5
0.4
0.3
0.2
0.1
0.0
0

ML (BRF)

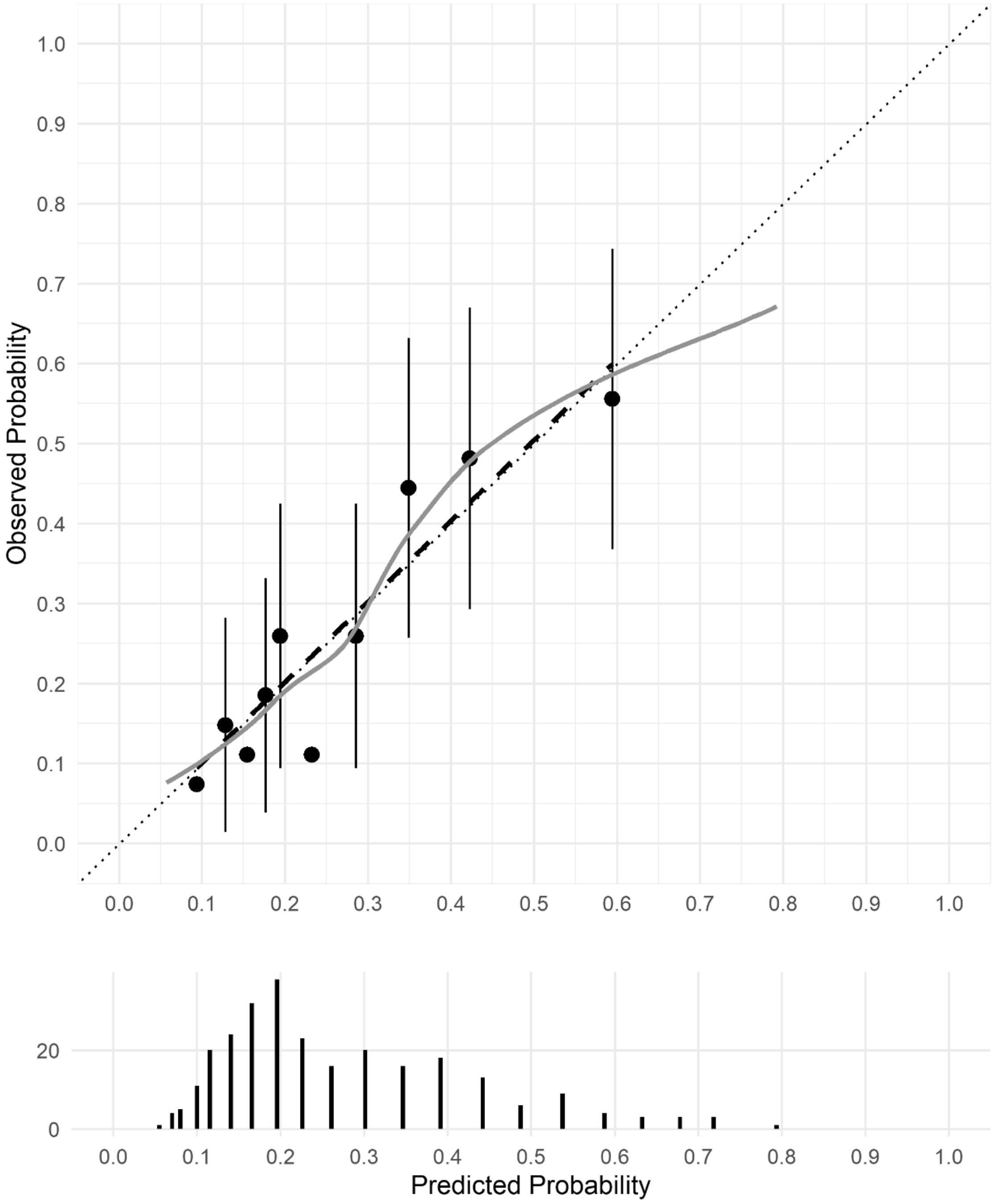
Observed Probability
Predicted Probability
0.0
0.1
0.2
0.3
0.4
0.5
0.6
0.7
0.8
0.9
1.0
0
20

SOFA score

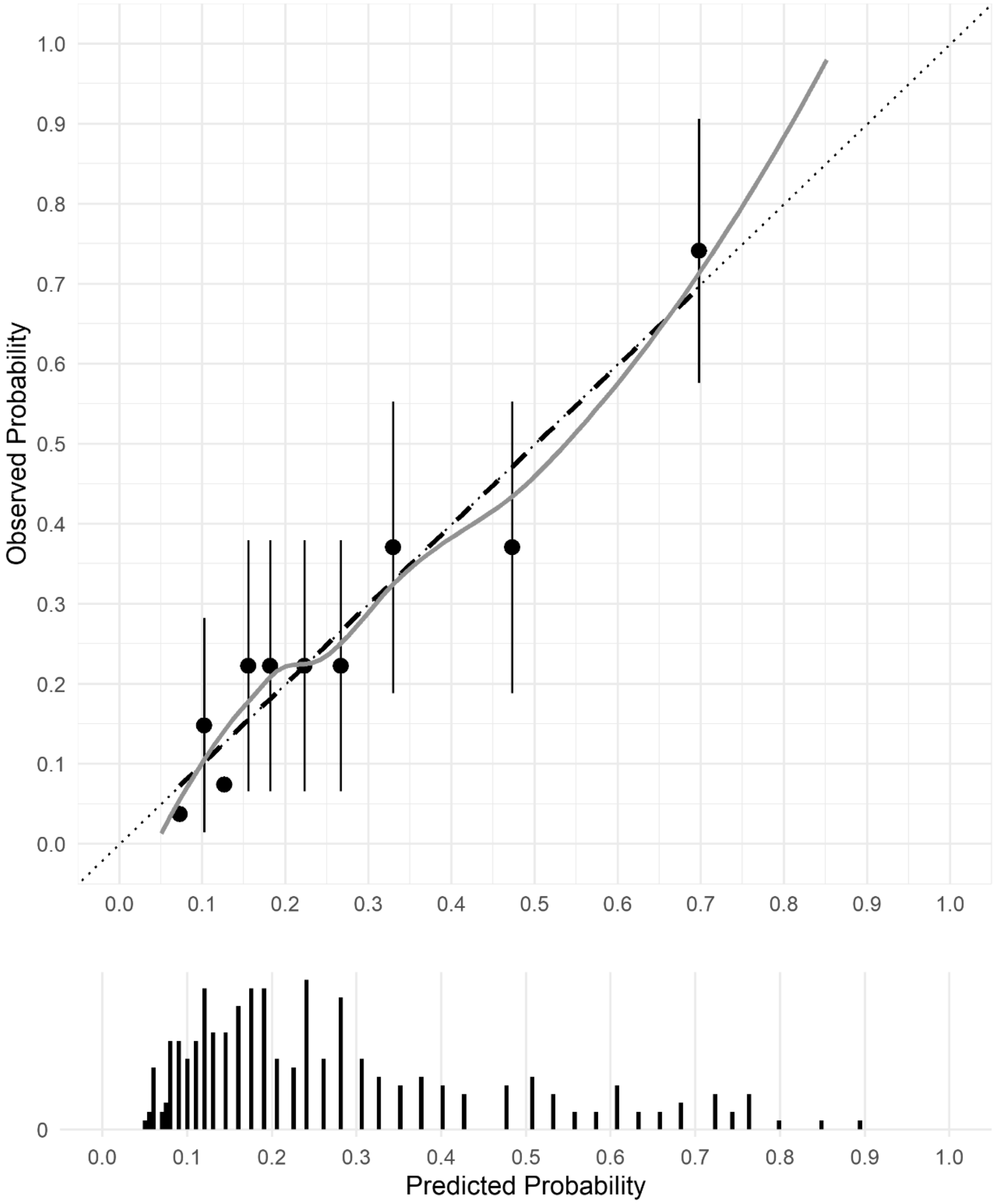

APACHE II score

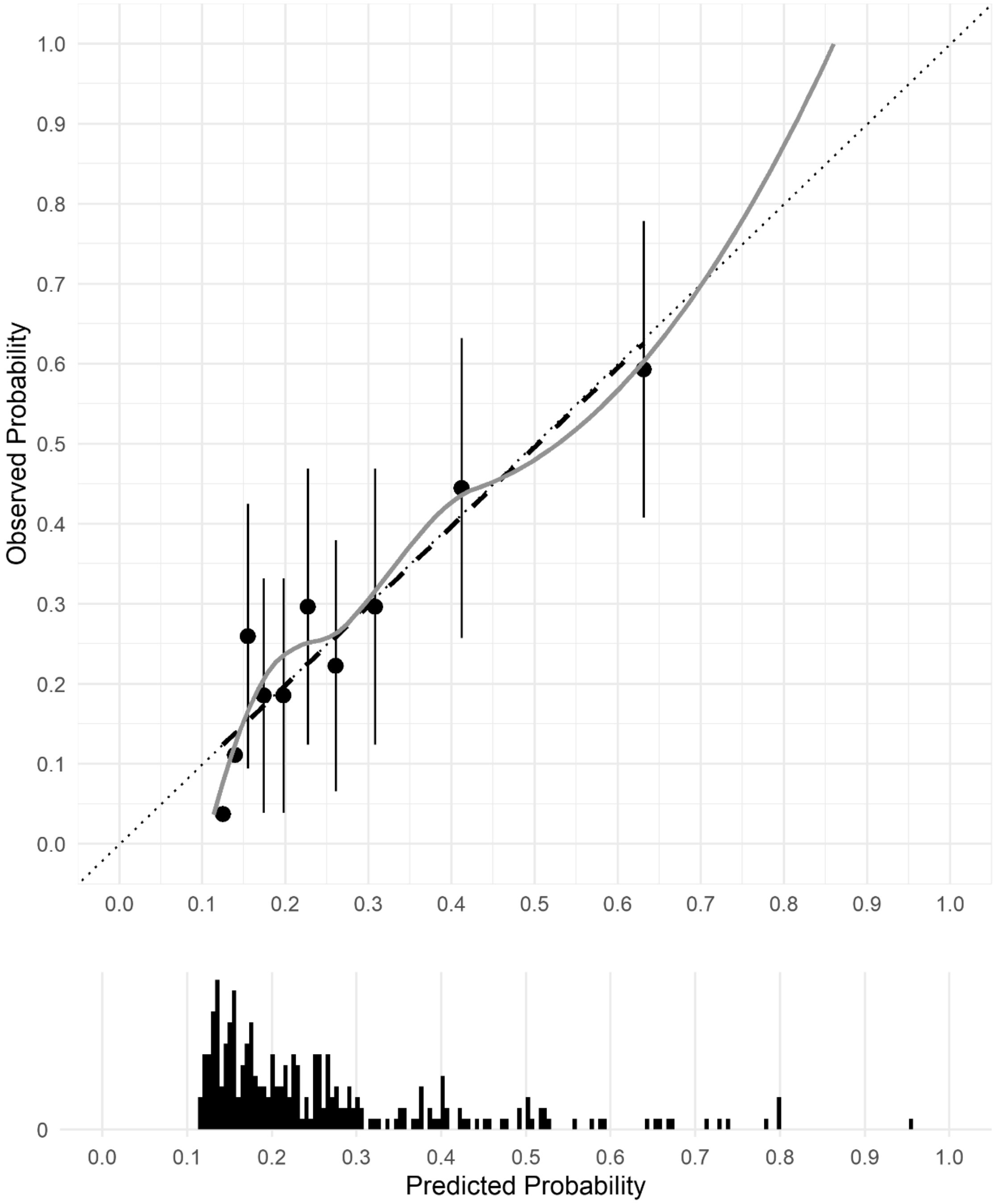

Initial lactate level
